# Gene-Based Association Analysis Identified Novel Genes Associated with Bone Mineral Density

**DOI:** 10.1371/journal.pone.0121811

**Published:** 2015-03-26

**Authors:** Xing-Bo Mo, Xin Lu, Yong-Hong Zhang, Zeng-Li Zhang, Fei-Yan Deng, Shu-Feng Lei

**Affiliations:** 1 Center for Genetic Epidemiology and Genomics, School of Public Health, Soochow University, Suzhou, Jiangsu, P. R. China; 2 Jiangsu Key Laboratory of Preventive and Translational Medicine for Geriatric Diseases, Soochow University, Suzhou, Jiangsu, P. R. China; 3 Department of Epidemiology, School of Public Health, Soochow University, Suzhou, Jiangsu, P. R. China; Central China Normal University, CHINA

## Abstract

Genetic factors contribute to the variation of bone mineral density (BMD), which is a major risk factor of osteoporosis. The aim of this study was to identify more “novel” genes for BMD. Based on the publicly available SNP-based *P* values, we performed an initial gene-based analysis in a total of 32,961 individuals. Furthermore, we performed differential expression, pathway and protein-protein interaction analyses to find supplementary evidence to support the significance of the identified genes. About 21,695 genes for femoral neck (FN)-BMD and 21,683 genes for lumbar spine (LS)-BMD were analyzed using gene-based association analysis. A total of 35 FN-BMD associated genes and 53 LS-BMD associated genes were identified (*P* < 2.3×10^-6^) after Bonferroni correction. Among them, 64 genes have not been reported in previous SNP-based genome-wide association studies. Differential expression analysis further supported the significant associations of 14 genes with FN-BMD and 19 genes with LS-BMD. Especially, *WNT3* and *WNT9B* in the Wnt signaling pathway for FN-BMD were further supported by pathway analysis and protein-protein interaction analysis. The present study took the advantage of gene-based association method to perform a supplementary analysis of the GWAS dataset and found some BMD-associated genes. The evidence taken together supported the importance of Wnt signaling pathway genes in determining osteoporosis. Our findings provided more insights into the genetic basis of osteoporosis.

## Introduction

Bone mineral density (BMD) is a major risk factor of osteoporosis and remains the best predictor of primary osteoporotic fractures[1]. Identification of genes predisposing to BMD will increase our understanding of its genetic mechanisms and contribute to development of novel prevention and treatment of osteoporosis and osteoporotic fractures.

Strong genetic factors contribute to the variation of BMD, with estimates of 84% heritability at femoral neck (FN) and 78% at lumbar spine (LS) [2]. In 2012, Estrada et al. reported the results of the largest effort to date searching for BMD-associated loci in > 80,000 subjects. They identified 56 loci associated with BMD at genome-wide significance (*P* < 5 × 10^-8^)[3]. Among these loci, 24 were previously reported [4–10], and 32 were new. However, the identified genes and their variants taken together explained only a small proportion (~ 6%) of total genetic variance in BMD [3]. It means that much more genetic factors need to be identified to explain the missing heritability of BMD.

Indeed, the traditional genome wide association studies (GWAS) ignored a large number of loci with moderate effects, due to the stringent significance thresholds adopted. Gene-based association analysis takes a gene as basic unit for association analysis. As this method can combine genetic information given by all the SNPs in a gene, it can obtain more informative results and increase the capability of finding novel genes. This method has been used as a novel complement method for SNP-based GWAS in identifying disease susceptibility genes[11]. This study presented a statistically robust gene-based association analysis, focusing on identifying “novel” genes for BMD.

## Material and Methods

### Study samples

The gene-based association study includes 32,961 individuals from the initial SNP-based GWAS conducted by the Genetic Factors of Osteoporosis consortium (GEFOS-2). Study design, subject characteristics, genotyping, data-quality filters and SNP-based association analysis were detailed in the original GWAS meta-analysis publication [3]. Briefly, it is a meta-analysis of multiple GWAS for FN-BMD (*n* = 32,961) and LS-BMD (*n* = 31,800 cases), including ~2.5 million genotyped or imputed autosomal SNPs from 17 studies of populations across North America, Europe, East Asia and Australia, with a variety of epidemiological designs and clinical characteristics of individuals. Subjects from 34 additional studies with BMD data (*n* = 50,933) were used for replication. This study identified 56 loci (32 new) associated with BMD at genome-wide significance (*P* < 5.0×10^-8^). The samples used in differential expression analyses included a total of 60 individuals from three studies. The effect of primary osteoporosis on the transcriptome of human mesenchymal stem cells (hMSC) from bone marrow was analyzed. Human MSC of 5 elderly patients suffering from osteoporosis were isolated from femoral heads after low-energy fracture of the femoral neck. Control cells were obtained from bone marrow of femoral heads of 9 non-osteoporotic donors after total hip arthroplasty[12]. Whole genome gene differential expression study of circulating monocytes was conducted in 12 subjects with extremely low peak bone mass and 14 subjects with extremely high peak bone mass [13]. Gene expression patterns of circulating B cells were compared in blood from 20 postmenopausal females with low (n = 10) or high (n = 10) BMD [14]. Details on study samples, quality control, experimental and data procedures were described in the original publications [12–14].

### Gene-based association analysis

Raw data used in the present gene-based association analysis were the downloaded *P* values, including FN-BMD and LS-BMD association *P* values of almost 2.5 million SNPs, which were publicly available (http://www.gefos.org/). Gene-based association analysis was performed using the GATES (Gene-based Association Test using Extended Simes procedure) method, which was modeled in the KGG software, a systematic biological Knowledge-based mining system for Genome-wide Genetic studies[11]. The extended Simes test integrated functional information and association evidence to combine the *P* values of the SNPs within a gene to obtain an overall *P* value for the association of the entire gene. This test is more powerful than the SNP-based test and does not require the raw genotype or phenotype data as inputs. It offers effective control of the type 1 error rate regardless of gene size and linkage disequilibrium (LD) pattern among markers, and does not need permutation or simulation to evaluate empirical significance. In the present gene-based association analysis, data files (for FN and LS-BMD association analysis) each contained four input variables, including the rs number, chromosome, position and SNP-based association *P* value of each SNP for KGG were prepared using the R program. The defined length of the extended gene region is from 2-kb upstream to 2-kb downstream of each gene. LD was adjusted based on CEU genotype data from HapMap release 2 in the analysis. To find whether any of the novel genes had been reported in previous association studies, we searched the databases of National Human Genome Research Institute (NHGRI, https://www.genome.gov/), GWAS Integrator (http://www.hugenavigator.net/HuGENavigator/) and Phenotype-Genotype Integrator (PheGenI, http://www.ncbi.nlm.nih.gov/gap/PheGenI/). Original GWAS publications were also reviewed. Bonferroni correction, the simplest and most conservative approach, was used to adjust for multiple testing[15].

### Differential expression analysis

Based on the normalized data available in the public databases (http://www.ncbi.nlm.nih.gov/geo), we tested differential expression of the identified BMD-associated genes by comparing mean gene expression signals in human mesenchymal stem cell (hMSC), monocytes or B cells between osteoporosis cases and controls using *t*-test. Four gene expression datasets were downloaded from gene expression omnibus (GEO) Database, namely, GSE35956 (hMSC), GSE35958 (hMSC), GSE7158 (monocytes) and GSE7429 (B cells). For the differential expression analyses, the significance level of *P* = 0.05 was used.

### Pathway analysis

We performed advanced gene set enrichment association (biological module-level association analysis) by using hybrid set-based test (HYST) [16]. The HYST combines all gene-based *P* values for association with correction of LD between genes. In the present pathway analysis, LD was adjusted based on CEU genotype data from HapMap release 2 in the analysis. The pathways were annotated according to the MsigDB database (http://www.broadinstitute.org/gsea/msigdb/), a secondary gene set database curated from KEGG, Reactome, Biocarta and the Pathway Interaction Database (PID). A total of 734 gene-sets were tested in the pathway analysis, the significance level for the pathway-based test was 6.8×10^-5^ according to the Bonferroni correction method.

### Protein-protein interaction network

To obtain functional evidence for the novel genes and evaluate the connectivity between these genes and BMD associated genes in the 56 loci confirmed in previous GWAS [3], we searched for protein-protein interaction networks in the STRING database (http://string-db.org/), a database of known and predicted protein interactions, including direct (physical) and indirect (functional) associations derived from genomic context, high throughput experiments, coexpression and previous knowledge (text mining) [17].

## Results

### Gene-based association analysis

**Fig 1:**
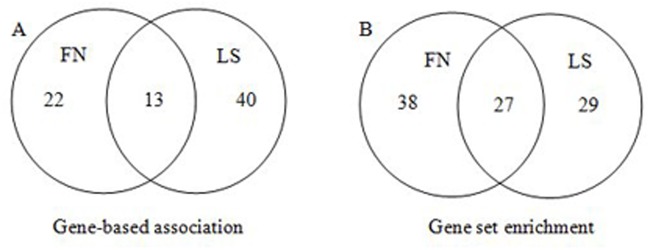
Venn diagram depicting genes and gene sets that were significantly associated with FN and LS-BMD. (A) In gene-based analyses, 35 genes were found to be associated with FN-BMD and 53 were associated with LS-BMD (*P* < 2.3×10^-6^), and 13 genes were overlapped; (B) In gene set enrichment analyses, there were in total 136 FN-BMD associated genes enriched in the 65 significant gene sets, and in total 151 genes enriched in the 56 significant gene sets, 27 gene sets were shared by FN-BMD and LS-BMD.

In the gene-based association analyses, 1,288,849 (49.8%) SNPs were mapped onto 21,695 genes on the human genome for FN-BMD and 1,283,560 (49.8%) SNPs were mapped onto 21,683 genes for LS-BMD. According to the Bonferroni correction method, the significance level for gene-based test was 2.3×10^-6^ for FN-BMD and LS-BMD. Accordingly, 75 significant genes were found for FN-BMD and LS-BMD. Among them, 35 were associated with FN-BMD (S1 Table), 53 were associated with LS-BMD (S2 Table), and 13 genes were overlapped (Fig. 1). If genes with *P* values just above the significance threshold were taken into account, 14 genes for FN-BMD (*GPRC5C*, *MEF2C-AS1*, *NSF*, *PTHLH*, *RAPGEF1*, *RC3H1*, *SMG6*, *SNORD67*, *SNORD91A*, *SNORD91B*, *SRR*, *TRAM1*, *TSR1*, *TTC21B-AS1*) and 6 genes for LS-BMD (*IGHMBP2*, *MTL5*, *POLR3A*, *RPS24*, *SKAP1*, *SPTBN1*) were site-specific. Within several of these genes the most associated variants did not reach the genome-wide significance threshold of 5.0×10^-8^. We searched the databases of PheGenI, NHGRI catalog and GWAS Integrator and compared the 75 genes with the list of previous identified genes with SNP-based *P* < 1.0×10^-5^. This comparison revealed that 11 genes were previously reported for association with BMD. The rest of 64 “novel” genes were first reported for BMD by the present study (S1 Table and S2 Table).

### Differential expression analysis

For the above 75 BMD-associated genes, we used *t*-test to compare mRNA expression signals in four expression studies. We found that 14 of the 35 FN-BMD-associated genes (Table 1) and 19 of the 53 LS-BMD-associated genes (Table 2) were differentially expressed in at least one study (*P* < 0.05). Several GWAS reported genes, such as *ZBTB40*, *ESR1*, *FGFRL1*, *CKAP5* and *SPTBN1*, were differentially expressed.

**Table 1:** Results of gene-based and differential expression analyses of FN-BMD associated genes.

Gene	*P*_gene	Chromosome	Reported^a^	*P*_expression			
				GSE35956	GSE35958	GSE7158	GSE7429
*ZBTB40*	1.11E-21	1	Y	4.96E-02	2.08E-02		
*SHFM1*	2.70E-18	7	N	1.33E-03			
*WNT3*	2.71E-10	17	N				2.88E-02
*WNT9B*	1.48E-09	17	N			4.10E-02	
*ESR1*	4.19E-09	6	Y	1.60E-03	7.71E-05		8.11E-04
*GPRC5C*	1.87E-07	17	N	4.20E-02			
*CKAP5*	4.75E-07	11	Y		1.42E-03		
*TSR1*	6.79E-07	17	N		3.44E-04		
*CD63*	9.19E-07	12	N	1.69E-03	1.89E-03		
*TRAM1*	1.30E-06	8	N		4.55E-03		
*RC3H1*	1.68E-06	1	N		1.61E-02		
*FGFRL1*	1.87E-06	4	Y	2.60E-02	2.00E-05		
*RAPGEF1*	1.97E-06	9	N	2.34E-02	3.30E-03		
*LOC286190*	2.39E-06	8	N	3.82E-02			

a: Y, genes have been reported in previous GWAS; N, genes have not been reported.

**Table 2:** Results of gene-based and differential expression analyses of LS-BMD associated genes.

Gene	*P*_gene	Chromosome	Reported^a^	*P*_expression			
				GSE35956	GSE35958	GSE7158	GSE7429
*ZBTB40*	1.99E-16	1	Y	4.96E-02	2.08E-02		
*SHFM1*	2.68E-11	7	N	1.33E-03			
*ESR1*	2.69E-11	6	Y	1.60E-03	7.71E-05		8.11E-04
*SPTBN1*	1.02E-09	2	Y	1.75E-02	5.38E-03		
*CD63*	3.83E-09	12	N	1.69E-03	1.89E-03		
*GAL*	9.78E-09	11	N		1.79E-02		
*ITGA7*	2.81E-08	12	N	1.57E-02	7.18E-04	1.92E-02	
*SARNP*	6.40E-08	12	N	9.51E-03			
*HOXB1*	3.34E-07	17	N		4.72E-02		
*HOXB9*	4.63E-07	17	N	3.78E-02			
*HOXB5*	5.26E-07	17	N	4.87E-03	1.86E-05		
*HOXB3*	5.88E-07	17	N	2.55E-02			
*RPS24*	6.57E-07	10	N	2.80E-02	1.08E-03		
*HOXB4*	8.43E-07	17	N	1.52E-02			
*LOC100134368*	1.02E-06	16	N	5.85E-05			
*IGHMBP2*	1.09E-06	11	N				4.61E-02
*ZNF652*	1.56E-06	17	N		2.63E-02		
*PHB*	2.01E-06	17	N	5.85E-03			
*BLOC1S1*	2.46E-06	12	N		1.80E-03		

a: Y, genes have been reported in previous GWAS; N, genes have not been reported.

### Pathway analysis

To gain insights into the functions of the identified genes, we tested the probability of significant genes (gene-based *P* < 0.05) clustering into a specific gene set defined by KEGG, Reactome, Biocarta or PID. Totally, 7,236 (33.4%) of the 21,695 gene-based analysis genes for FN-BMD and 7,233 (33.4%) of the 21,683 gene-based analysis genes for LS-BMD have been registered in the pathway datasets. For FN-BMD, 136 genes (gene-based *P* < 0.05, duplicates removed) were enriched in 65 pathways, and 10 genes (*ESR1*, *WNT3*, *WNT9B*, *SMG6*, *TNFRSF11B*, *SHFM1*, *F2*, *RPE65*, *RAPGEF1*, *CD63*) with gene-based *P* < 2.3×10^-6^ were involved in these pathways (S3 Table). For LS-BMD, 151 genes (gene-based *P* < 0.05, duplicates removed) were enriched in 56 significant pathways, and 7 genes (*ESR1*, *TNFRSF11B*, *CPT1A*, *RPE65*, *SPTBN1*, *SHFM1*, *ITGA7*) with gene-based *P* < 2.3×10^-6^ were involved in these pathways (S4 Table). There were 27 pathways shared by FN-BMD and LS-BMD (Fig. 1). Enrichments in the well-known Wnt signaling (*P* = 2.65×10^-56^), cell carcinoma (*P* = 2.12×10^-81^), drug metabolism (*P* = 7.94×10^-33^), hormone biosynthesis (*P* = 2.04×10^-13^), TGFBR (*P* = 3.49×10^-31^), MAPK signaling (*P* = 7.01×10^-33^) pathways were observed. Those pathways are all important in the biology and etiology of BMD and osteoporosis.

### Protein-protein interactions

**Fig 2:**
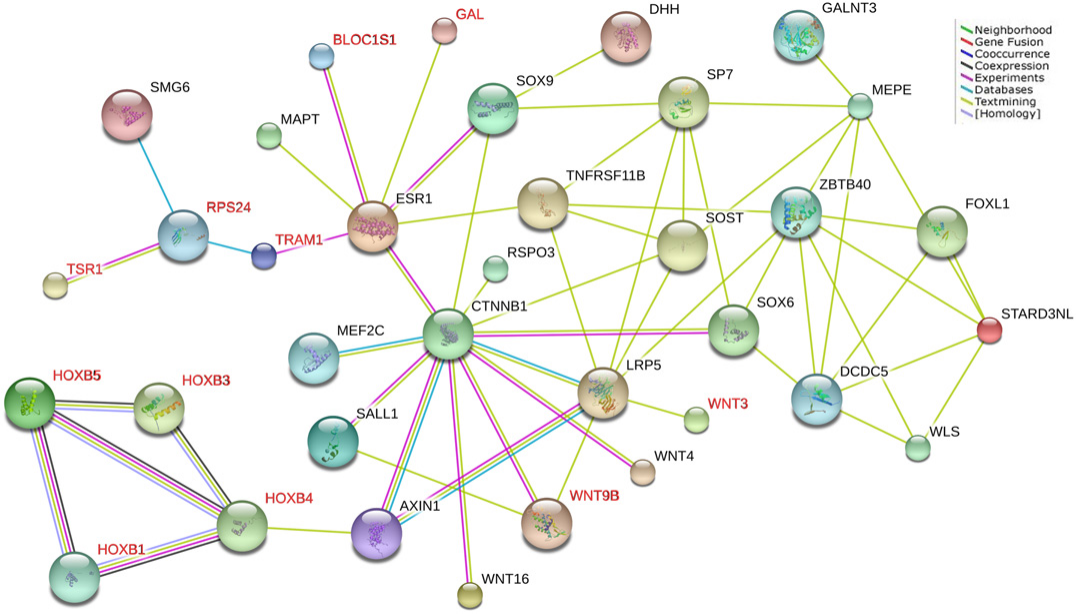
Protein-protein interactions between novel and previously-identified BMD-associated genes. Novel and previously-identified BMD-associated genes which interacted with each other in the network were presented. Red colored gene symbols indicated genes newly identified (eleven genes), while black indicated previous identified genes.

We tested the protein-protein interactions between the 24 newly identified (supported by the expression data) and the 56 BMD associated genes reported in the original GWAS in the STRING database. Eleven genes, including 4 *HOXB* cluster genes (*HOXB1*, *HOXB3*, *HOXB4* and *HOXB5*), *WNT3* and *WNT9B* in the Wnt signaling pathway, *TSR1*, *RPS24*, *TRAM1*, *BLOC1S1* and *GAL*, were found to be connected with each other or with the GWAS identified genes (Fig. 2).

## Discussion

This gene-based association study identified 64 BMD-associated genes. Differential expression studies further supported the significant association of some of the identified genes. Pathway analysis and protein-protein interaction analysis gave further evidence on several novel BMD associated genes. The evidence taken together supported the importance of *WNT3* and *WNT9B* in the Wnt signaling pathway in determining osteoporosis.

During the recent 7 years, GWAS have revolutionized the understanding of the genetic architecture of complex diseases and traits. In a large scale GWAS meta-analysis, 56 loci that influence BMD variation have been confirmed [3]. Moreover, nearly 200 genes or loci associated with BMD (*P* < 1.0×10^-5^) have been reported (by searching the NHGRI, PheGenI and GWAS Integrator databases). These findings have provided important insights into bone biology and the mechanisms of osteoporosis. However, with a stringent significance thresholds adopted, many moderate association signals in GWAS datasets were ignored. Besides, GWAS always focused on the most significant variants when replicating the associations and reported gene regions. In regions which contain large stretches of LD containing dozens of genes in the same haplotype block, many important genes would be missed. Gene-based association analysis methods have been proposed and considered to be a robust complement method to identify disease susceptibility genes in GWAS. In the present study, we used the GATES method to analyze the published data of the large scale GWAS meta-analysis [3]. As expected, we identified 64 new genes that were associated with FN or LS BMD, including *WNT3* and *WNT9B* in the Wnt signaling pathway.

Not surprisingly, the identified genes tend to enrich in bone-associated pathways, especially in the well-known and very important Wnt signaling pathway. Twelve genes with a gene-based *P* value less than 0.05 were enriched in the KEGG Wnt signaling pathway, and two of them (*WNT3* and *WNT9B*) were significantly associated with FN-BMD after multiple testing correction. Protein-protein interaction analysis showed that *WNT3* and *WNT9B* both interacted with *LRP5* (encoding the low-density lipoprotein receptor-related protein 5), that is a node of Wnt signaling pathway. The *LRP5* gene has been reported to affect bone mass accrual during growth and cause high bone density [18,19]. Several Wnt genes have been reported to be associated with BMD in previous GWAS, including *CTNNB1*, *SOST*, *LRP4*, *LRP5*, *WLS*, *WNT4*, *MEF2C*, *WNT5B*, *WNT16*, *DKK1*, *PTHLH*, *SFRP4* and *AXIN1*. The most associated variants within *WNT3* and *WNT9B* have reached the genome-wide significance threshold in the GWAS of GEFOS consortium, but these genes were not reported in the original publication [3]. The present study adds *WNT3* and *WNT9B* to this list of important bone-influencing Wnt factors.

The homeobox genes encode a highly conserved family of transcription factors that play an important role in morphogenesis in all multicellular organisms. Mammals possess four similar homeobox gene clusters, *HOXA*, *HOXB*, *HOXC* and *HOXD*. *HOXC6* have been reported to be associated with BMD in the original GWAS [3]. Genes in *HOXB* cluster encoded proteins functioned as sequence-specific transcription factors that were involved in development, cell proliferation and differentiation. *HOXB1* and *HOXB2* were reported to be associated with primary tooth development in GWAS [20]. *HOXB4* and *HOXB5* were associated with myelomonocytic leukemia [21,22]. *HOXB3* and *HOXB5* were associated with obesity[23]. Interaction between the *HOXB* cluster genes was observed, and *HOXB4* was interacted with *AXIN1*, a gene involved in the Wnt signaling pathway. Although the connection was weak, but the relationship between *HOXB4* and cell intrinsic pathway genes have been proven. Indeed, *HOXB4* has been shown to influence Wnt signaling at multiple stages [24]. These findings highlight the importance of the *HOXB* cluster genes in the etiology of osteoporosis. However, these findings are needed to be tested in future studies.

*ESR1* gene plays an important role in bone growth and maintenance of bone mass by regulating metabolism and acquisition of peak bone mass as well as inhibiting bone loss. Different polymorphisms have been described in the *ESR1* gene [25]. The association between *ESR1* and BMD has also been confirmed by GWAS[3]. The present study also supported this association, including results from gene-based analysis, differential expression analysis, pathway analysis and protein-protein interaction analysis. More importantly, several BMD-associated genes, including *GAL*, *BLOC1S1*, *TSR1*, *TRAM1* and *RPS24*, showed connection with *ESR1* in protein-protein interaction analysis. Although the bone related function of these genes is unknown, the findings suggested the importance of these genes in the etiology of osteoporosis.

A significant genetic correlation of BMD at the FN and LS has been reported [26], suggesting that there are pleiotropic genetic factors in determining BMD at the FN and LS. As we expected, the present study found some significant genes and pathways shared by FN-BMD and LS-BMD. On the other hand, we also found some skeletal site-specific BMD-associated genes and pathways, suggesting differential genetic influences on BMD determination across skeletal sites, as previous study pointed out[3].

This gene-based association study confirmed the importance of many genes that were identified in previous SNP-based studies, but missed some reported genes, which included two major cases. In one case, the association signals of some of the missed reported genes have been detected, but the signals are only suggestive (*P* values from 3.18×10^-4^ to 2.3×10^-6^) and very closed to significant cutoff (*P* = 2.3×10^-6^). The gene-based association takes all SNPs in the gene into account, and the *P* value reflects the effect of the entire gene. In this situation, even though the gene comprises SNP(s) that achieve the genome wide significance level, the gene-based *P* value would not necessarily reach the significance level. The other case is that some significant signals of intergenic SNPs may not be mapped onto a gene (note that only 49.5% of the SNPs were mapped onto genes) in the gene-based analysis. These signals were missed in the analysis.

The definition of gene region length was from 2-kb upstream to 2-kb downstream in our gene-based analysis. This definition may lead to miss the SNPs with significant signals in the gene-based association analysis if they are located closely at but outside of the defined gene regions. Additionally, this definition may cover only a nearest promoter, and thus miss the point of regulation, enhancers and distal promoters, and other non-genic regulome variants. However, this parameter seems not so critical in the analysis. We found that the results changed slightly when using different settings, such as from 5-kb upstream to 5-kb downstream for each gene.

The purpose of this study is to get meaningful biologically important information out of publicly available data by applying robust statistical and bioinformatics analysis. The gene-based association analysis is a very important complement method for SNP-based GWAS. The current gene-based analysis is similar to a previous study [27] in the strategy. However, some remarked differences and obvious improvements existed between the two studies. For example, the sample size in previous study is only about 6,700, but more than 32,000 in the current study. The gene-based analysis model is using the VEGAS in the previous study [27] but the GATES in our study. Additional analyses (e.g., differential expression analysis, protein-protein interaction analysis) were performed. As we expected, this gene-based GWAS confirmed several BMD genes and also detected several novel BMD genes.

According to our pathway analysis, enrichments of the detected genes in the well-known Wnt signaling, cell carcinoma, drug metabolism, hormone biosynthesis, TGFBR, MAPK signaling pathways were observed. Those pathways are all important in the biology and etiology of BMD and osteoporosis. The interactions between the identified genes and the well-known bone-related genes suggested the importance of these genes in the etiology of bone metabolism and osteoporosis. However, although we found supplementary functional information to support the significant findings, it is still unclear how much of the extent of these genes attributable to bone formation and loss, and whether they are population dependent. The biological verification for the findings is still limited, so further studies were needed to validate the associations and elucidate the mechanisms.

In conclusion, the present study took the advantage of gene-based association methods to perform a supplementary analysis of the GWAS dataset and found some novel FN and LS-BMD associated genes. The expression analysis, pathway analysis and protein-protein interaction analysis gave supportive evidence for the gene-based association analysis discoveries. Our findings provided more insights into the genetic basis of BMD.

## Supporting Information

S1 Table Results of gene-based analysis of FN-BMD associated genes. (PDF)

S2 Table Results of gene-based analysis of LS-BMD associated genes. (PDF)

S3 Table Results of gene set enrichment analysis of FN-BMD associated genes. (PDF)

S4 Table Results of gene set enrichment analysis of LS-BMD associated genes. (PDF)
