# Supplementary material for: Gene-Based Association Analysis Identified Novel Genes Associated with Bone Mineral Density
Source: PLoS One. 2015 Mar 26;10(3):e0121811. doi: 10.1371/journal.pone.0121811 (PMC4374695; doi:10.1371/journal.pone.0121811)
Supplement: S1 Table — (PDF) [file pone.0121811.s001.pdf]

Table S1 Results of gene-based analysis of FN-BMD associated genes

Gene	*P* _gene	Chromosome	Locus	Reported_gene*
ZBTB40	1.11E-21	1	1p36.12	ZBTB40
LOC100506136	1.34E-18	7	7q21.3	SLC25A13
SHFM1	2.70E-18	7	7q21.3	SLC25A13
MEF2C-AS1	3.72E-18	5	5q14.3	MEF2C
TNFRSF11B	5.50E-15	8	8q24	TNFRSF11B
CCDC170	3.02E-13	6	6q25.1	C6orf97
RPE65	6.03E-11	1	1p31	WLS
WNT3	2.71E-10	17	17q21	C17orf53
WNT9B	1.48E-09	17	17q21	C17orf53
ESR1	4.19E-09	6	6q25.1	C6orf97
SCN1A	5.75E-09	2	2q24.3	GALNT3
TTC21B	1.48E-08	2	2q24.3	GALNT3
ENOX1	7.13E-08	13	13q14.11	AKAP11
MIR5582	9.49E-08	11	11p11.2	ARHGAP1
F2	1.74E-07	11	11p11.2	ARHGAP1
GPRC5C	1.87E-07	17	17q25	N
FBLN5	2.45E-07	14	14q32.1	MARK3
SNORD67	3.80E-07	11	11p11.2	ARHGAP1
ARL17B	4.52E-07	17	17q21.31	C17orf53
CKAP5	4.75E-07	11	11p11.2	ARHGAP1
SNORD91A	6.03E-07	17	17p13.3	SMG6
SNORD91B	6.03E-07	17	17p13.3	SMG6
TSR1	6.79E-07	17	17p13.3	SMG6
GDF11	6.89E-07	12	12q13.2	SMG6
SRR	7.91E-07	17	17p13.3	SMG6
CD63	9.19E-07	12	12q12-q13	DHH
TTC21B-AS1	1.07E-06	2	2q24.3	GALNT3
NSF	1.07E-06	17	17q21	C17orf53
PTHLH	1.16E-06	12	2p12.1-p11.	KLHDC5
TRAM1	1.30E-06	8	8q13.3	XKR9
RC3H1	1.68E-06	1	1q25.1	N
FGFRL1	1.87E-06	4	4p16	IDUA
RAPGEF1	1.97E-06	9	9q34.3	FUBP3
SMG6	2.06E-06	17	17p13.3	SMG6
NSFP1	2.29E-06	17	17q21	C17orf53
LOC286190	2.39E-06	8		N
LOC100134368	3.11E-06	16		N
OR6C1	3.12E-06	12		N
LACTB2	3.35E-06	8		N
SARNP	3.40E-06	12		N
ITGA7	3.63E-06	12		N
SGSM2	4.00E-06	17		N
TMEM204	4.09E-06	16		N
RERE	4.49E-06	1		Y
XKR9	4.72E-06	8		Y
DCDC5	5.26E-06	11		Y

SPARCL1	5.28E-06	4	N
GAL	5.52E-06	11	N
LRRC37A	7.62E-06	17	N
TMEM8A	8.01E-06	16	N
OR6C3	8.36E-06	12	N
NME4	1.12E-05	16	N
MEF2C	1.19E-05	5	Y
OR6C6	1.23E-05	12	N
EML4	1.24E-05	2	N
MIR9-2	1.30E-05	5	N
KIF19	1.48E-05	17	N
LRP4	1.66E-05	11	Y
IFT140	1.74E-05	16	N
LRP4-AS1	1.75E-05	11	N
DMP1	1.88E-05	4	N
ACP2	1.91E-05	11	N
PIWIL3	2.17E-05	22	N
PHB	2.62E-05	17	N
LINC00461	2.84E-05	5	N
OR6C74	2.90E-05	12	N
ZNF408	3.59E-05	11	Y
TRMT61B	4.08E-05	2	N
ARFGAP2	4.08E-05	11	N
ESYT2	4.28E-05	7	N
DDB2	4.58E-05	11	N
SLC9C1	4.80E-05	3	N
PRAC1	4.89E-05	17	N
FAM210A	4.98E-05	18	Y
GPR142	5.01E-05	17	N
PRAC2	5.07E-05	17	N
HOXB13	5.22E-05	17	N
MIR3185	5.22E-05	17	N
C11orf49	5.35E-05	11	N
HOXB8	5.39E-05	17	N
HOXB1	5.93E-05	17	N
BTBD17	5.96E-05	17	N
SNF8	6.04E-05	17	N
HOXB9	6.44E-05	17	N
MIR196A1	6.61E-05	17	N
PACSIN3	7.36E-05	11	N
ZNF652	7.36E-05	17	N
HOXB2	7.39E-05	17	N
HOXB7	7.57E-05	17	N
TMEM87B	7.80E-05	2	N
DCAF7	7.92E-05	17	N
ZNF45	7.99E-05	19	N
MTL5	8.09E-05	11	N
IPP	8.26E-05	1	N

HOXB6	9.07E-05	17	N
HOXB-AS1	9.44E-05	17	N
KCNH6	9.47E-05	17	N
HOXB5	9.56E-05	17	N
HOXB3	1.03E-04	17	N
HOXB-AS3	1.08E-04	17	N
GLS	1.16E-04	2	N
NUDT9	1.19E-04	4	N
GCSAM	1.21E-04	3	N
SOX6	1.24E-04	11	Y
ACE	1.34E-04	17	N
OR6C75	1.37E-04	12	N
SERPINC1	1.38E-04	1	N
PRKAG2	1.40E-04	7	N
CDAN1	1.42E-04	15	N
NEUROD4	1.47E-04	12	N
TRIP11	1.56E-04	14	N
TESPA1	1.65E-04	12	N
OVOL1	1.68E-04	11	N
HOXB4	1.77E-04	17	N
MIR10A	1.77E-04	17	N
CD200	1.81E-04	3	N
MIR765	1.84E-04	1	N
UBE2Z	1.88E-04	17	N
RNMT	1.91E-04	18	N
TTLL6	2.06E-04	17	N
SPDYA	2.20E-04	2	N
MIR4707	2.25E-04	14	N
PPM1J	2.39E-04	1	N
CALCOCO2	2.41E-04	17	N
LRRC71	2.45E-04	1	N
TC2N	2.48E-04	14	N
CPT1A	2.62E-04	11	N
PSMC5	2.74E-04	17	N
FTSJ3	2.76E-04	17	N
MIR3649	2.87E-04	12	N

*: Y, genes have been reported in previous GWAS; N, genes have not been reported.
