# Supplementary material for: Gene-Based Association Analysis Identified Novel Genes Associated with Bone Mineral Density
Source: PLoS One. 2015 Mar 26;10(3):e0121811. doi: 10.1371/journal.pone.0121811 (PMC4374695; doi:10.1371/journal.pone.0121811)
Supplement: S2 Table — (PDF) [file pone.0121811.s002.pdf]

Table S2 Results of gene-based analysis of LS-BMD associated genes

Gene	*P* _gene	Chromosome	Locus	Reported_gene*
ENOX1	1.70E-28	13	13q14.11	AKAP11
CCDC170	6.30E-18	6	6q25.1	C6orf97
TNFRSF11B	7.12E-17	8	8q24	TNFRSF11B
ZBTB40	1.99E-16	1	1p36.12	ZBTB40
RPE65	2.30E-14	1	1p31	WLS
SHFM1	2.68E-11	7	7q21.3	SLC25A13
ESR1	2.69E-11	6	6q25.1	C6orf97
LOC100506136	4.25E-11	7	7q21.3	SLC25A13
POLR3A	1.54E-10	10	10q22-q23	KCNMA1
MTL5	5.72E-10	11	11q13.2-q13.3	LRP5
SPTBN1	1.02E-09	2	2p21	PKDCC
CPT1A	1.21E-09	11	11q13.3	LRP5
FBLN5	1.56E-09	14	14q32.1	MARK3
NEUROD4	1.85E-09	12	12q13.2	SP7
CD63	3.83E-09	12	12q12-q13	DHH
GDF11	4.44E-09	12	12q13.2	SP7
GAL	9.78E-09	11	11q13.3	SP7
ITGA7	2.81E-08	12	12q13.2	SP7
MRPL21	6.17E-08	11	11q13.3	SP7
SARNP	6.40E-08	12	12q13.2	SP7
TESPA1	7.67E-08	12	12q13.2	SP7
ARL17B	1.57E-07	17	17q21.31	C17orf53
PRAC1	3.17E-07	17	17q21	C17orf53
HOXB8	3.20E-07	17	17q21.3	C17orf53
PRAC2	3.29E-07	17	17q21	C17orf53
HOXB1	3.34E-07	17	17q21.3	C17orf53
HOXB13	3.38E-07	17	17q21.2	C17orf53
MIR3185	3.38E-07	17	17q21.32	C17orf53
HOXB2	3.64E-07	17	17q21.32	C17orf53
HOXB6	4.55E-07	17	17q21.32	C17orf53
HOXB-AS1	4.58E-07	17	17q21.32	C17orf53
HOXB9	4.63E-07	17	17q21.32	C17orf53
HOXB7	4.73E-07	17	17q21.32	C17orf53
MIR196A1	5.21E-07	17	17q21.32	C17orf53
HOXB5	5.26E-07	17	17q21.32	C17orf53
HOXB-AS3	5.60E-07	17	17q21.32	C17orf53
HOXB3	5.88E-07	17	17q21.32	C17orf53
TRIP11	5.93E-07	14	14q31-q32	MARK3
RPS24	6.57E-07	10	10q22	KCNMA1
SCN1A	7.10E-07	2	2q24.3	GALNT3
HOXB4	8.43E-07	17	17q21.3	MAPT
MIR10A	8.43E-07	17	17q21.3	MAPT
DSPP	9.92E-07	4	4q21.3	MEPE
LOC100134368	1.02E-06	16	16p13.3	AXIN1
IGHMBP2	1.09E-06	11	11q13.3	LRP5
TTLL6	1.13E-06	17	17q21.32	C17orf53

CALCOCO2	1.40E-06	17	17q21.32	C17orf53
ZNF652	1.56E-06	17	17q21.32	C17orf53
DCDC5	1.57E-06	11	11p14.1	DCDC5
SKAP1	1.69E-06	17	17q21	C17orf53
TC2N	1.95E-06	14	14q32.12	RPS6KA5
PHB	2.01E-06	17	17q21.32	C17orf53
OR6C6	2.03E-06	12	12q13.2	DHH
BLOC1S1	2.46E-06	12		N
TMEM8A	2.61E-06	16		N
LOC400654	2.70E-06	18		N
BLOC1S1-RDH5	3.68E-06	12		N
RDH5	3.68E-06	12		N
DMP1	3.70E-06	4		N
UBE2Z	5.40E-06	17		N
NSFP1	5.80E-06	17		N
MIR3649	6.25E-06	12		N
CBX1	7.66E-06	17		N
MPP7	7.87E-06	10		Y
SNF8	8.15E-06	17		N
FGFRL1	8.61E-06	4		Y
OR6C75	8.71E-06	12		N
PDXDC1	8.87E-06	16		Y
LOC284294	1.10E-05	18		N
SNX11	1.16E-05	17		N
OR6C3	1.21E-05	12		N
OR6C1	1.31E-05	12		N
NME4	1.41E-05	16		N
FAM3C	1.44E-05	7		Y
WNT16	1.56E-05	7		Y
FOSL1	1.69E-05	11		N
LRRC37A	1.90E-05	17		N
ATF1	2.59E-05	12		N
OR6C74	2.61E-05	12		N
SAMD12-AS1	3.24E-05	8		N
RRN3	3.46E-05	16		N
NFE2L1	3.46E-05	17		N
EPDR1	3.57E-05	7		Y
RIC8A	4.31E-05	11		N
LINC00305	4.79E-05	18		N
WNT3	5.15E-05	17		N
CTBP1-AS	6.08E-05	4		N
TSGA10IP	6.19E-05	11		N
CTSW	6.62E-05	11		N
NLRP6	6.74E-05	11		N
LOC100130872	7.10E-05	4		N
FIBP	7.94E-05	11		N
SIRT3	8.08E-05	11		N
TTC21B	9.72E-05	2		N

HARBI1	1.01E-04	11	N
SNRPA	1.05E-04	19	N
ATG13	1.07E-04	11	N
MIR5582	1.09E-04	11	N
SATB2-AS1	1.11E-04	2	N
ARFGAP2	1.16E-04	11	N
MIA	1.35E-04	19	N
IQCE	1.35E-04	7	N
ANKRD63	1.50E-04	15	N
SNORA55	1.57E-04	1	N
EGLN2	1.61E-04	19	N
ACP2	1.79E-04	11	N
DNAJC24	1.83E-04	11	N
ARHGAP1	1.91E-04	11	Y
ZNF408	1.91E-04	11	Y
SATB2	1.95E-04	2	N
C2orf16	1.99E-04	2	N
REM2	2.02E-04	14	N
PACSIN3	2.02E-04	11	N
STOX1	2.03E-04	10	N
CTBP1	2.03E-04	4	N
DIP2B	2.12E-04	12	N
MIR4688	2.25E-04	11	N
MRPL28	2.42E-04	16	N
ZNF512	2.42E-04	2	N
AMBRA1	2.42E-04	11	N
DECR2	2.43E-04	16	N
PPIEL	2.43E-04	1	N
C6orf211	2.46E-04	6	N
F2	2.59E-04	11	Y
LIN7C	2.61E-04	11	Y
TTLL11	2.62E-04	9	N
MIA-RAB4B	2.69E-04	19	N
RAB4B	2.69E-04	19	N
SUMO1	2.77E-04	2	N
PPM1J	2.79E-04	1	N
BMP8A	2.79E-04	1	N
WNT9B	2.85E-04	17	N
NT5C1A	2.90E-04	1	N
OR6C65	2.95E-04	12	N
MIR548AA1	2.96E-04	9	N
MIR548D1	2.96E-04	9	N
DDB2	3.14E-04	11	N
TMEM198B	3.18E-04	12	N

*: Y, genes have been reported in previous GWAS; N, genes have not been reported.
