# Supplementary material for: Gene-Based Association Analysis Identified Novel Genes Associated with Bone Mineral Density
Source: PLoS One. 2015 Mar 26;10(3):e0121811. doi: 10.1371/journal.pone.0121811 (PMC4374695; doi:10.1371/journal.pone.0121811)
Supplement: S3 Table — (PDF) [file pone.0121811.s003.pdf]

Table S3 Results of gene set enrichment analysis of FN-BMD associated genes

GeneSet	*P*_HYST	GeneSet_Gene #	No. of Gene_*P*<0.05	Significant Gene	*P*_gene
BIOCARTA_CARM_ER_PATHWAY	2.608E-93	35	4	ESR1	4.19496E-09
PID_P38ALPHABETADOWNSTREAMPATHWAY	2.712E-90	38	3	ESR1	4.19496E-09
KEGG_BASAL_CELL_CARCINOMA	2.122E-81	55	6	WNT3	2.709E-10
-	-	-	-	WNT9B	1.48185E-09
PID_TELOMERASEPATHWAY	4.313E-78	68	6	ESR1	4.19496E-09
-	-	-	-	SMG6	2.0596E-06
PID_HDAC_CLASSII_PATHWAY	2.782E-73	34	5	ESR1	4.19496E-09
PID_WNT_SIGNALING_PATHWAY	5.671E-64	28	2	WNT3	2.709E-10
BIOCARTA_ALK_PATHWAY	1.471E-60	37	2		
BIOCARTA_HDAC_PATHWAY	9.213E-60	32	4		
KEGG_WNT_SIGNALING_PATHWAY	2.648E-56	151	12	WNT3	2.709E-10
-	-	-	-	WNT9B	1.48185E-09
BIOCARTA_NFAT_PATHWAY	2.731E-51	56	7	F2	1.7402E-07
BIOCARTA_LEPTIN_PATHWAY	6.316E-47	11	2		
PID_AP1_PATHWAY	5.608E-45	70	9	ESR1	4.19496E-09
KEGG_MELANOGENESIS	9.033E-42	102	9	WNT3	2.709E-10
-	-	-	-	WNT9B	1.48185E-09
KEGG_RETINOL_METABOLISM	3.017E-38	64	19	RPE65	6.025E-11
PID_ER_NONGENOMIC_PATHWAY	8.023E-37	41	4	ESR1	4.19496E-09
KEGG_CYTOKINE_CYTOKINE_RECEPTOR_INTERACTION	8.786E-37	267	12	TNFRSF11B	5.49556E-15
KEGG_MAPK_SIGNALING_PATHWAY	7.006E-33	267	19		
KEGG_DRUG_METABOLISM_CYTOCHROME_P450	7.942E-33	72	17		
PID_ATF2_PATHWAY	1.087E-32	59	6	ESR1	4.19496E-09
KEGG_METABOLISM_OF_XENOBIOTICS_BY_CYTOCHROME_P450	4.922E-29	70	15		
REACTOME_NUCLEAR_RECEPTOR_TRANSCRIPTION_PATHWAY	3.088E-25	49	3	ESR1	4.19496E-09
REACTOME_XENOBIOTICS	7.155E-24	16	8		
REACTOME_BIOLOGICAL_OXIDATIONS	4.555E-21	139	17		
KEGG_PROTEASOME	1.319E-19	48	4	SHFM1	2.7042E-18
REACTOME_GLUCURONIDATION	1.36E-17	18	8		
KEGG_STARCH_AND_SUCROSE_METABOLISM	9.007E-16	52	10		
KEGG_DRUG_METABOLISM_OTHER_ENZYMES	2.933E-14	51	14		
KEGG_ASCORBATE_AND_ALDARATE_METABOLISM	6.381E-14	25	9		
KEGG_PENTOSE_AND_GLUCURONATE_INTERCONVERSIONS	1.534E-13	28	9		

KEGG_STEROID_HORMONE_BIOSYNTHESIS	2.039E-13	55	13		
REACTOME_CYTOCHROME_P450_ARRANGED_BY_SUBSTRATE_TYPE	8.761E-13	51	9		
PID_SYNDECAN_4_PATHWAY	9.741E-12	32	3	F2	1.7402E-07
REACTOME_PHASE_II_CONJUGATION	9.931E-12	70	8		
PID_THROMBIN_PAR4_PATHWAY	2.916E-11	15	3	F2	1.7402E-07
REACTOME_PHASE1_FUNCTIONALIZATION_OF_COMPOUNDS	4.749E-11	70	9		
SIG_CHEMOTAXIS	2.242E-10	45	4		
KEGG_PORPHYRIN_AND_CHLOROPHYLL_METABOLISM	3.89E-10	41	10		
REACTOME_PLATELET_AGGREGATION_PLUG_FORMATION	4.525E-10	36	4	F2	1.7402E-07
REACTOME_GLUCONEOGENESIS	1.44E-09	34	4		
KEGG_FOCAL_ADHESION	2.893E-09	201	11	RAPGEF1	1.96988E-06
PID_AMB2_NEUTROPHILS_PATHWAY	8.51E-09	41	6		
REACTOME_CYTOKINE_SIGNALING_IN_IMMUNE_SYSTEM	1.762E-08	270	20	RAPGEF1	1.96988E-06
PID_IL1PATHWAY	2.602E-08	34	4		
BIOCARTA_IL1R_PATHWAY	2.371E-07	33	3		
PID_IL23PATHWAY	6.337E-07	37	3		
KEGG_LEISHMANIA_INFECTION	0.000001018	72	3		
PID_ANGIOPOIETINRECEPTOR_PATHWAY	0.000002457	50	4	F2	1.7402E-07
REACTOME_IL1_SIGNALING	0.000004688	39	2		
BIOCARTA_CELL2CELL_PATHWAY	0.000007909	14	1		
BIOCARTA_EXTRINSIC_PATHWAY	0.00001059	13	2	F2	1.7402E-07
REACTOME_COMMON_PATHWAY	0.00001186	14	2	F2	1.7402E-07
KEGG_HYPERTROPHIC_CARDIOMYOPATHY_HCM	0.00002499	85	7		
BIOCARTA_RHO_PATHWAY	0.00004803	32	3		
BIOCARTA_CYTOKINE_PATHWAY	0.0001929	22	2		
REACTOME_ARMS_MEDIATED_ACTIVATION	0.0007265	17	4	RAPGEF1	1.96988E-06
REACTOME_SIGNALING_BY_ILS	0.0028	107	8	RAPGEF1	1.96988E-06
REACTOME_PLATELET_ACTIVATION_SIGNALING_AND_AGGREGATION	0.003774	208	11	F2	1.7402E-07
-	-	-	-	CD63	9.186E-07
PID_ECADHERIN_NASCENTAJ_PATHWAY	0.004554	39	4	RAPGEF1	1.96988E-06
REACTOME_REGULATION_OF_IFNG_SIGNALING	0.005497	14	2		
PID_NCADHERINPATHWAY	0.006254	33	3		
PID_IFNGPATHWAY	0.006333	40	6	RAPGEF1	1.96988E-06
PID_SHP2_PATHWAY	0.01096	58	5		
KEGG_LEUKOCYTE_TRANSENDOTHELIAL_MIGRATION	0.01373	118	8		
SA_TRKA_RECEPTOR	0.01723	17	4		
KEGG_NEUROTROPHIN_SIGNALING_PATHWAY	0.01983	126	16	RAPGEF1	1.96988E-06

“P_HYST” is the P value of the hybrid set-based test.
