# Supplementary material for: Gene-Based Association Analysis Identified Novel Genes Associated with Bone Mineral Density
Source: PLoS One. 2015 Mar 26;10(3):e0121811. doi: 10.1371/journal.pone.0121811 (PMC4374695; doi:10.1371/journal.pone.0121811)
Supplement: S4 Table — (PDF) [file pone.0121811.s004.pdf]

Table S4 Results of gene set enrichment analysis of LS-BMD associated genes

GeneSet	*P*_HYST	GeneSet_Gene#	No. of Gene_*P*<0.05	Significant Gene	*P*_gene
BIOCARTA_LEPTIN_PATHWAY	2.43E-113	11	3	CPT1A	1.21E-09
PID_MYC_PATHWAY	4.33E-82	25	6		
MIPS_STAGA_COMPLEX	1.11E-81	13	3		
PID_AP1_PATHWAY	4.63E-56	70	8	ESR1	2.69E-11
PID_HDAC_CLASSII_PATHWAY	1.16E-53	34	4	ESR1	2.69E-11
PID_NOTCH_PATHWAY	5.75E-52	59	8		
REACTOME_SIGNALING_BY_NOTCH	2.11E-51	103	8		
KEGG_CYTOKINE_CYTOKINE_RECEPTOR_INTERACTION	7.58E-51	267	24	TNFRSF11B	7.12E-17
KEGG_BASAL_CELL_CARCINOMA	2.14E-47	55	8		
REACTOME_NUCLEAR_RECEPTOR_TRANSCRIPTION_PATHWAY	1.73E-46	49	3	ESR1	2.69E-11
KEGG_RETINOL_METABOLISM	1.92E-35	64	19	RPE65	2.30E-14
KEGG_ENDOMETRIAL_CANCER	3.73E-32	52	5		
PID_TGFBRPATHWAY	3.49E-31	55	6	SPTBN1	1.02E-09
REACTOME_CTNNB1_PHOSPHORYLATION_CASCADE	3.76E-26	16	3		
PID_NCADHERINPATHWAY	4.36E-26	33	5		
PID_WNT_SIGNALING_PATHWAY	6.27E-26	28	4		
KEGG_DRUG_METABOLISM_CYTOCHROME_P450	7.57E-26	72	21		
BIOCARTA_PITX2_PATHWAY	8.76E-26	15	2		
KEGG_HEMATOPOIETIC_CELL_LINEAGE	5.76E-25	88	9		
KEGG_WNT_SIGNALING_PATHWAY	1.06E-23	151	17		
BIOCARTA_WNT_PATHWAY	6.19E-23	26	4		
BIOCARTA_PS1_PATHWAY	1.84E-22	14	2		
PID_BETACATENIN_DEG_PATHWAY	5.38E-22	18	3		
KEGG_PROTEASOME	4.55E-21	48	3	SHFM1	2.68E-11
BIOCARTA_GSK3_PATHWAY	1.31E-20	27	4		
PID_WNT_CANONICAL_PATHWAY	5.86E-20	20	2		
PID_ILK_PATHWAY	1.34E-17	45	6		
REACTOME_SIGNALING_BY_NOTCH4	3.17E-16	12	2		
IGAND_BINDING_INITIATES_THE_SECOND_PROTEOLYTIC_CLEAVAG	1.41E-15	12	2		
REACTOME_CLASS_B_2_SECRETIN_FAMILY_RECEPTORS	1.80E-15	88	10		
PID_IFNGPATHWAY	4.16E-14	40	4		
KEGG_DRUG_METABOLISM_OTHER_ENZYMES	2.41E-13	51	16		

KEGG_TYPE_I_DIABETES_MELLITUS	2.96E-13	44	5		
MIPS_DSS1_COMPLEX	4.01E-13	13	2	SHFM1	2.68E-11
PID_PS1PATHWAY	8.62E-13	46	4		
KEGG_ASCORBATE_AND_ALDARATE_METABOLISM	8.86E-13	25	9		
BIOCARTA_ALK_PATHWAY	3.89E-12	37	6		
PID_THROMBIN_PAR4_PATHWAY	1.19E-11	15	3		
PID_CONE_PATHWAY	1.27E-11	23	5	RPE65	2.30E-14
KEGG_INTESTINAL_IMMUNE_NETWORK_FOR_IGA_PRODUCTION	1.86E-10	48	7		
EGG_ARRHYTHMOGENIC_RIGHT_VENTRICULAR_CARDIOMYOPATHY_ARV	4.13E-10	76	5	ITGA7	2.81E-08
PID_HDAC_CLASSI_PATHWAY	8.01E-10	66	6		
KEGG_STEROID_HORMONE_BIOSYNTHESIS	2.31E-09	55	12		
KEGG_ALLOGRAFT_REJECTION	4.71E-09	38	5		
PID_SHP2_PATHWAY	1.70E-06	58	8		
KEGG_HYPERTROPHIC_CARDIOMYOPATHY_HCM	4.70E-06	85	8	ITGA7	2.81E-08
BIOCARTA_IL1R_PATHWAY	1.12E-05	33	3		
KEGG_GRAFT_VERSUS_HOST_DISEASE	2.56E-05	42	4		
BIOCARTA_IL5_PATHWAY	4.24E-05	10	3		
PID_SYNDECAN_4_PATHWAY	7.23E-05	32	3		
REACTOME_REGULATION_OF_IFNG_SIGNALING	1.19E-03	14	2		
BIOCARTA_NKT_PATHWAY	1.25E-03	29	5		
PID_AMB2_NEUTROPHILS_PATHWAY	1.53E-03	41	8		
REACTOME_PLATELET_AGGREGATION_PLUG_FORMATION	3.97E-03	36	4		
PID_NFAT_TFPATHWAY	5.43E-03	47	4		
REACTOME_RAP1_SIGNALLING	1.16E-02	17	3		

“P_HYST” is the P value of the hybrid set-based test.
